# Buckwheat Hulls/Perlite as an Environmentally Friendly Flame-Retardant System for Rigid Polyurethane Foams

**DOI:** 10.3390/polym15081913

**Published:** 2023-04-17

**Authors:** Strąkowska Anna, Miedzińska Karolina, Członka Sylwia

**Affiliations:** Institute of Polymer and Dye Technology, Faculty of Chemistry, Lodz University of Technology, 90-537 Lodz, Poland

**Keywords:** polyurethane foams, buckwheat hulls, perlite, flame-retardant

## Abstract

This article presents an innovative approach to the flame retardancy of rigid polyurethane foams using natural waste in the form of buckwheat hulls in combination with an inorganic additive—perlite. A series of tests were presented in which various contents of flame-retardant additives were used. Based on the test results, it was found that the addition of the buckwheat hull/perlite system affected the physical and mechanical properties of the obtained foams, i.e., apparent density, impact strength, and compressive and flexural strength. The structure of the system had also changed, directly affecting the hydrophobic properties of the foams. In addition, it was observed that the addition of buckwheat hull/perlite modifiers improved the burning behavior of composite foams.

## 1. Introduction

Polyurethanes are obtained in a step-by-step polyaddition reaction of compounds containing two or more isocyanate groups (di- or triisocyanates), with compounds containing hydroxyl (polyols) or amino groups to form a urethane bond in their main chain with the structure [-O-CO-NH-] [1]. Polyurethane materials are a very important group among all polymers because they account for approximately 8% of the world’s production of plastics. Foamed polyurethane plastics are widely used as construction materials in the furniture or automotive industry and in construction as thermal insulation elements, among other uses [1,2,3,4]. A special position among commonly used polyurethanes is occupied by flexible, semi-rigid, and rigid polyurethane foams, which account for approximately two-thirds of their global production.

The weak point of polyurethane foams from a usability point of view is their flammability. Not only do they ignite from a small fire source but they burn at a high speed. During their combustion, a great deal of heat is released and smoke and toxic gases are produced. The thermal decomposition of the polyurethane composite occurs at temperatures above 200 °C. The decomposition products are primarily hydrogen cyanide and carbon monoxide but nitrogen oxides, nitriles, hydrogen chloride, and carbon dioxide are also present. The simplest compounds are formed at temperatures exceeding 800 °C as a result of the defragmentation of previous decomposition products [5,6,7,8]. For this reason, much attention has been paid to reducing the flammability of PU foams. Currently, flame retardant properties can be improved by adding flame retardants. The selection of an effective flame-retardant substance for polymer composites requires the fulfillment of appropriate requirements. The desired properties effectively prevent fires and thus do not cause the emission of smoke or toxic substances during combustion [5]. Until recently, the use of halogen flame retardants was popular; however, due to the release of large amounts of smoke and toxic gases into the atmosphere during their combustion, these additives have been abandoned. Therefore, out of concern for environmental protection, research on halogen-free flame retardants as flame-retardant compounds for PU foams has been undertaken. One of the known flame retardants of polymeric materials is expandable graphite [9,10,11,12]. However, graphite is not always suitable for polymerization molding as it results in the faster wear of foam injection equipment. Therefore, more attention is being paid to the search for other alternative flame retardants that will be both environmentally friendly and economically viable. Waste biomass seems to be an interesting source that can be used to enrich the properties of polyurethane composites, making them more bio-friendly at the same time. Due to their chemical composition, buckwheat hulls seem to be an attractive, potentially flame-retardant raw material for the production of PU foams. Buckwheat is often called a phosphorus absorber because it can absorb more phosphorus from the soil than other plants. Hence, the chemical composition of the scales includes numerous phosphorus compounds in addition to calcium, magnesium, and potassium compounds [13]. The production of buckwheat generates approximately three tons of husk waste per one ton of groats. Approximately 30% of buckwheat hulls are used for the production of pillows, fuel pellets, and briquettes, and the remainder is permanently stored. Therefore, it is important to find a use for the significant quantities of stored shells. However, due to their organic nature, buckwheat hulls require a conjugated additive to support their flame-retardant effect [14]. Perlite is an amorphous volcanic glass with very low density and high porosity. It consists mainly of SiO_2_, Al_2_O_3_, Na_2_O, K_2_O, and water [15]. It is also characterized by high thermal stability and when heated to a temperature of 760 to 1100 °C, it increases its volume by up to 7–16 times and acquires the properties of a thermal and acoustic insulator [16,17]. Due to its low thermal conductivity and good fire resistance, perlite seems to be a potentially good flame-retardant modifier [18].

The results obtained in this study indicate that the addition of buckwheat hulls and perlite in any amount affects the morphology and properties of modified PU foams primarily by effectively reducing their flammability.

## 2. Materials

The water-blown rigid PU foams used in this study were obtained from a two-component system supplied by *Purinova* sp. z o. o. (Bydgoszcz, Poland) after mixing the polyol (Izopianol 30/10/C) and diphenylmethane diisocyanate (Purocyn B). Polyol is a mixture of components containing polyester polyol (hydroxyl number ca. 230–250 mg KOH/g, functionality of 2), a catalyst (N,N-Dimethylcyclohexylamine), flame retardant properties (Tris(2-chloro-1-methylethyl) phosphate), a chain extender (1,2-propanediol), and water as blowing agent. Buckwheat hulls were supplied by a local company from Poland and perlite powder with a specific surface of 60–70 m^2^/g was supplied by Sigma-Aldrich (Saint Louis, MO, USA).

## 3. Manufacturing of Rigid PU Foams

**Figure 1:**
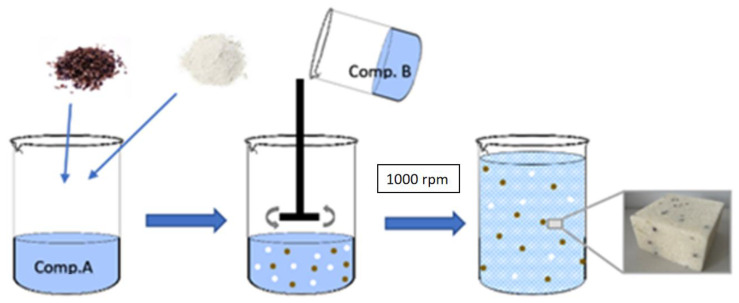
Method of preparing PU foams.

Rigid PU foams containing buckwheat hulls and perlite as flame retardants were obtained by adding the appropriate number of modifiers to Izopianol 30/10/C and then the mixture (component A) was homogenized with an overhead mixer at 1000 RPM in ambient conditions for approximately 60 s. Purocyn B (component B) was added to component A and the mixture was mixed for 15 s at 1800 rpm. According to the information provided by the supplier, the components were mixed at a ratio of 100:160 (ratio of component A to component B). The prepared system was poured into an open mold and allowed to expand freely in a vertical direction. The scheme for preparing PU foams is shown in Figure 1.

Rigid PU foams were conditioned at room temperature for 24 h. After this time, the samples were cut with a band saw into appropriate shapes and their physical and mechanical properties were tested. The composition of individual systems is shown in Table 1.

## 4. Methods

The morphology and cell size distribution of foams were examined from cellular structural images of foam, which were taken using ESEM Quanta 250 FEG scanning electron microscopy (FEI Company, Hillsboro, OR, USA). The samples were tested in low vacuum mode (80 Pa) using an LFD detector. Pictures were taken in five areas of each foam at different zoom intensities. Sample surfaces were imaged. The average pore diameter, wall thickness, and pore size distribution were calculated using ImageJ software (Media Cybernetics Inc., Rockville, MD, USA).

The apparent density of foams was determined according to ASTM D1622 (equivalent to ISO 845). The densities of five samples were measured and averaged.

The compressive strength (σ10%) of foams was determined according to the ASTM D1621 (equivalent to ISO 844) using a Zwick Z100 testing machine (Zwick/Roell Group, Ulm, Germany) with a load cell of 2 kN and speed of 2 mm min^−1^. Samples of the specified sizes were cut with a band saw in a direction perpendicular to the foam growth direction. Then, the analyzed sample was placed between two plates and the compression strength was measured as a ratio of the load, causing a 10% deformation of the sample cross-section in the parallel and perpendicular directions to the square surface. The result was averaged for five measurements per sample.

The three-point bending test was carried out using a Zwick Z100 testing machine (Zwick/Roell Group, Germany) at room temperature in accordance with ASTM D7264 (equivalent to ISO 178). The samples were bent at a speed of 2 mm min^−1^. The results of the bending stress at break (εf) are presented as the average values obtained for five samples in a series.

The hardness of polyurethane foams was determined by the Shore method in accordance with the ISO 868 standard. A Shore hardness tester (type 00) by Zwick/Roell, equipped with a ball indenter with a diameter of 1.2 mm, was used during the experiment. During the testing of each series of foams, at least 15 measurements were taken. The results are presented as average values.

The nature of the surface was analyzed by measuring the contact angle for distilled water (drop volume was set to: 1 μL). Before the measurement, the surfaces of the samples were dedusted. OCA A 15EC goniometer from DataPhysics InstrumentsGmbH (Filderstadt, Germany) was used, equipped with a single direct dosing system (0.01-1 ml B. Braunsyringe, Hassen, Germany). The presented results are the average value of at least 10 measurements of drops on the surface of the foams.

The water absorption of rigid PU foams was measured according to ASTM D2842. First, the foams were dried for 1 h at 80 °C and then their mass was checked. Then, the samples were immersed in distilled water to a depth of 1 cm for 24 h and then removed from the water. Excess water was blotted off with tissue paper and the foams were weighed again. The results are presented as the mean values of five measurements.

Thermal stability tests of the rigid PU foams were carried out using the TGA/DSC1 thermogravimetric analyzer from Mettler Toledo. Thermal decomposition was carried out in the range of 25–800 °C in an inert gas atmosphere at a flow rate of 50 mL/min and a heating rate of 10 °C/min. The measurements were based on examining the change in the mass of the sample as a function of temperature. In order to analyze the sample decomposition process, decomposition temperatures were determined—T5%, T10%, and T50%—at which specific mass losses were noted.

The thermal conductivity of the obtained foams (λ) was determined. The test was carried out for samples with dimensions of 200 mm × 200 mm× 20 mm using a FOX 200 apparatus (LaserComp Inc., Livonia, MI, USA). Thermal conductivity was determined at 10, 20, and 40 °C in the range of 20 to 100 mW/(m*K).

The flammability of rigid PU foams was determined using a cone calorimeter in accordance with the ISO 5660 standard at S.Z.T.K, “TAPS”—Maciej Kowalski (Lodz, Poland). Each sample with dimensions of 100 × 100 × 25 mm^3^ was wrapped in aluminum foil and burned at an external heat flux of 35 kW m^−2^. At that time, the parameters of the combustion process were recorded, i.e., ignition time (IT), total heat release (THR), total smoke release (TSR), the average yields of CO and CO_2_ (COY and CO_2_Y), and the maximum average rate of heat emission (MAHRE). The measurement for each sample was repeated three times; the results given are the averages of the three measurements.

## 5. Results and Discussion

### 5.1. Structure of the Rigid PU Foams

**Figure 2:**
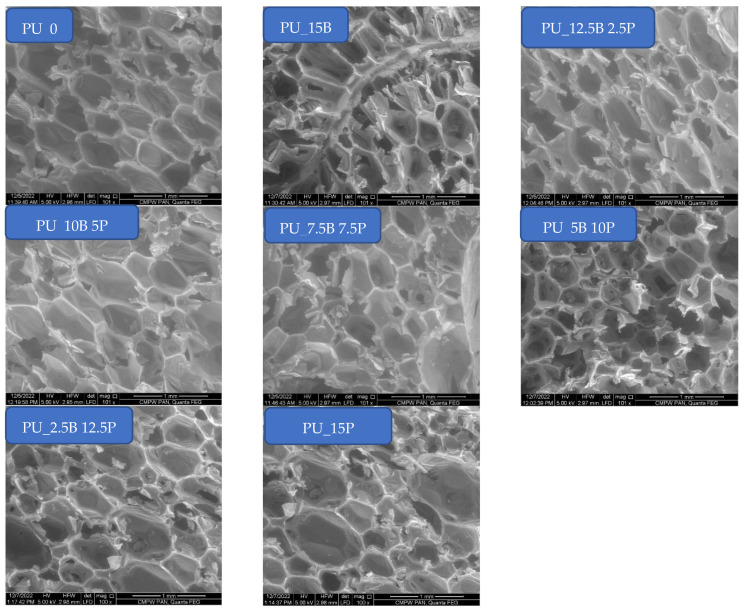
SEM images of rigid PU foams containing buckwheat hulls and perlite in different amounts.

Rigid PU foams were prepared using the bucket method, in which modifiers were first added to component A (polyol) in appropriate amounts and then mixed for 1 min to obtain the best possible homogenization. Depending on the amount of each additive, the viscosity of the system changed—this has been repeatedly tested in previous works [19,20,21]. The higher viscosity of the system translates into a change in structure—it becomes more porous with smaller cells. This was also the case with the introduction of buckwheat hulls and perlite in the amount of 15 parts by weight in various proportions. The structures obtained in individual compositions are shown in Figure 2.

**Figure 3:**
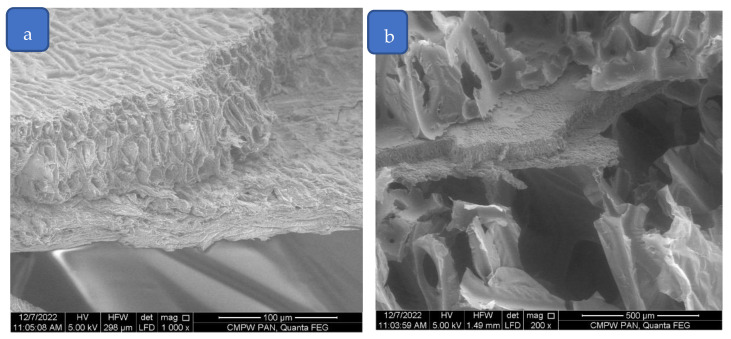
SEM images of buckwheat hull structure (**a**) and buckwheat hulls building into the structure of rigid PU foam (**b**).

The above pictures show that the standard foam without additives is characterized by the greatest regularity. The addition of buckwheat and, in particular, perlite, affects the structure disorder, whereby with an increasing amount of perlite, the structure becomes more heterogeneous. The cells are more differentiated in terms of size and the cell anisotropy characteristic of polyurethane foams disappears. This phenomenon is caused by the structure and particle size of the modifiers. Buckwheat hulls are much larger and build into the structure of the foam, interfering only in the rheology of the system at close range. In addition, the rough structure of the buckwheat surface favors a good connection with the porous PU composite, which can be observed in Figure 3. On the other hand, dusty perlite with much smaller fragmentation mainly affects the system by changing its viscosity, causing disturbances in the structure due to the hindered expansion of bubbles during foam growth.

Changes in structure as a result of the modifications are well illustrated by the pore sizes in the obtained foams, presented in Table 2. Based on the results, it can be observed that with the addition of flame retardants, pore size distribution significantly increases, which is particularly visible for high perlite contents. Furthermore, this addition also increases the thickness of the walls between the pores, which may indicate the deposition of perlite particles in them.

### 5.2. Mechanical Properties

**Figure 4:**
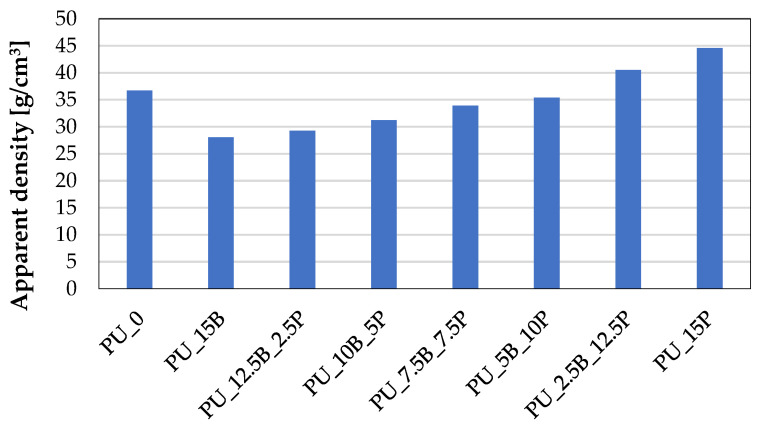
Apparent density of rigid PU foams containing buckwheat hulls and perlite in different amounts.

Such a variable structure of the foams has an impact on their usable properties, including mechanical ones. On the one hand, a more heterogeneous structure tends to weaken the foam due to the presence of more defects in the form of large and often open cells. On the other hand, small particles of the modifier can build into the intercellular spaces—the so-called ribs—allowing for additional reinforcement of the structure. When buckwheat hulls and perlite were introduced into the polyurethane composite, a change in the structure could also be observed in different apparent densities of the obtained foams (Figure 4). The addition of buckwheat hulls to the foams reduced the apparent density from 37 g/cm^3^ to 28 g/cm^3^ in comparison to the reference foam PU_0. This could be influenced by the low density of buckwheat hulls, which were well incorporated into the foam structure. However, as a result of introducing perlite to the system, a gradual increase in the apparent density of the foams was observed that was proportional to the content of perlite. Pearlite particles, despite their low density, likely favored the occlusion of the reaction mixture during synthesis, and the foams themselves were characterized by a smaller pore size, which was the result of a higher viscosity of the system. The foam with the highest content of perlite PU_15P was characterized by an apparent density of 44 g/cm^3^.

**Figure 5:**
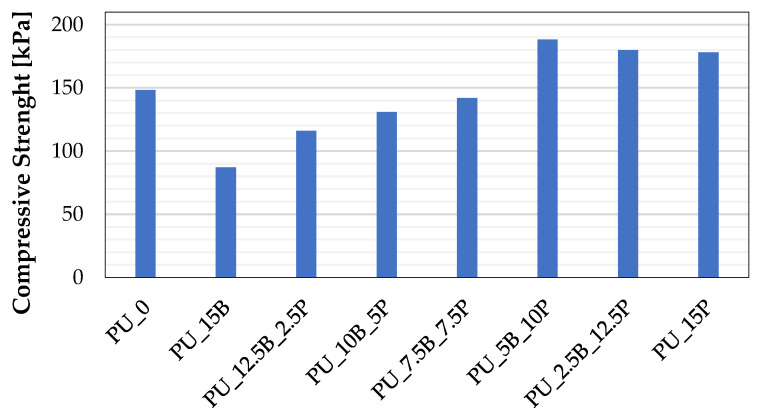
Compression strength of rigid PU foams containing buckwheat hulls and perlite.

Differences in structure and apparent densities were also reflected in the mechanical properties of the foams—their compressive and flexural strength. In Figure 5, a similar strength tendency can be observed for PU composites resulting from the content of modifiers. The addition of buckwheat hulls weakened the compressive strength measured in the direction parallel to the direction of foam growth, which may have been caused by the lower density of the foams and the presence of large pores, which were more easily subjected to the pressure of force and were destroyed during the measurement. In turn, the increasing content of pearlite strengthened the structure by the more accurate incorporation of particles in the intercellular spaces and by reducing the size of the pores. Foams with a high content of perlite (above 10 parts by weight) were characterized by much better mechanical properties (over 20% improvement) than the reference foam PU_0.

**Figure 6:**
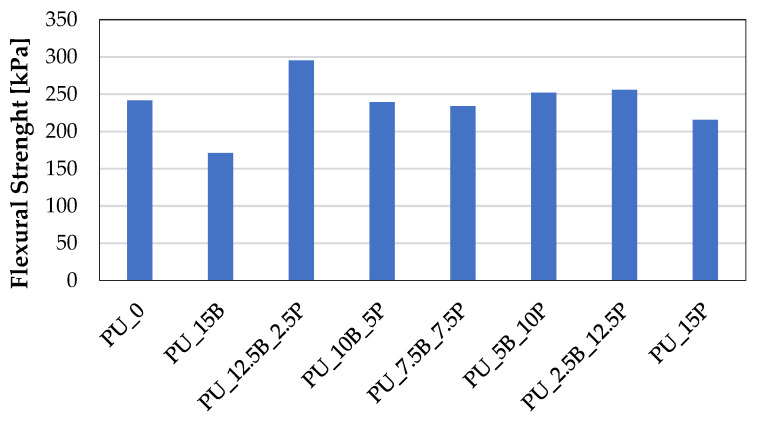
Flexural strength of rigid PU foams containing buckwheat hulls and perlite.

In the case of bending strength, a clear tendency could not be observed. Due to the large dispersion of pore sizes and the heterogeneous structure of the composites filled with buckwheat husks/perlite, the foam properties determined during three-point bending were characterized by large discrepancies and low repeatability. Nevertheless, based on the obtained results presented in Figure 6, it can be concluded that these properties were at a similar level to those of the reference foam, and the introduction of additional substances into the composite did not affect the deterioration of the bending resistance.

**Figure 7:**
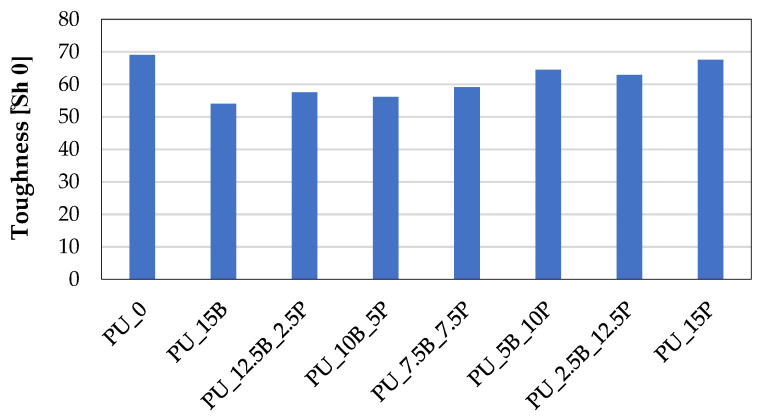
Toughness of rigid PU foams containing buckwheat hulls and perlite.

The hardness of the foam after modification was also measured where, as a result of the introduction of additional substances resulting in a decrease in the homogenization of the structure, the hardness decreased (Figure 7). This decrease was slightly more significant in the case of composites with a higher content of buckwheat hulls and decreased due to the addition of perlite. However, all the composites containing the flame-retardant additive were characterized by lower hardness than the reference sample PU_0.

### 5.3. Water-Related Properties

The water-related properties of composite foams were assessed using water uptake and contact angle methods. The water absorption ability of materials characterized by a porous structure depends mostly on their cell structure (whether the cells are open or closed) and hydrophilic-hydrophobic nature.

**Figure 8:**
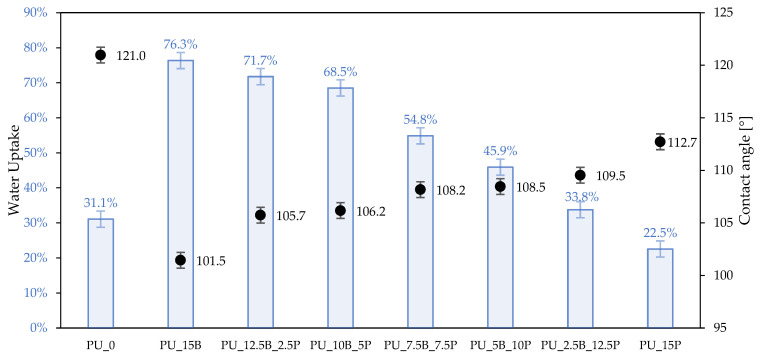
Water-related properties of rigid PU foams—water uptake and contact angle results.

**Figure 9:**
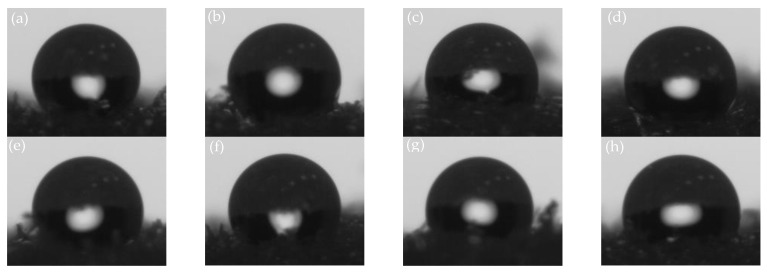
Images of contact angle examination for (**a**) PU_0, (**b**) PU_15B, (**c**) PU_12.5B_2.5P, (**d**) PU_10B_5P, (**e**) PU_7.5B_7.5P, (**f**) PU_5B_10P, (**g**) PU_2.5B_12.5P, and (**h**) PU_15P.

As presented in Figure 8, the addition of buckwheat hulls/perlite modifiers noticeably changed the water absorption capacity and contact angle values of analyzed polyurethane foams. Compared with the unmodified foam PU_0, the water uptake increased with increases in the buckwheat husks content (the highest value was achieved by PU_15B and was 76.3%) and decreased with increases in the perlite content, achieving a water uptake value (22.5%) for the PU_15P foam that was lower than that for pure, unmodified foam, for which the water uptake value was 31.1%. In the case of contact angles, the highest value (121.0°) was observed for the reference foam. The foams with the addition of modifiers showed the values of the contact angles in accordance with the relationship that the higher the content of buckwheat husks (and the lower content of perlite filler), the lower the contact angle values. The values of the contact angles of the modified foams decreased from 112.7° for PU_15P to 101.5° for PU_15B. This may be due to various reasons, one of which may be the hydrophilicity of buckwheat husks, which increases the water absorption of foams [22]. In order to better illustrate the change in the surface character as a result of the addition of buckwheat/perlite husks, Figure 9. presents images of water drops on the surface of the composites. In the case of the reduced water uptake of foams with the addition of perlite, this may be related to the higher density of these foams (lower sorption capacity) and the physical barrier created by perlite particles embedded in the structure. Foams with perlite were characterized by a more heterogeneous structure, with a large share of cells of smaller size than the reference foam, which favors greater hydrophobicity and more difficulty in penetrating the structure by water.

### 5.4. Thermal Stability

**Figure 10:**
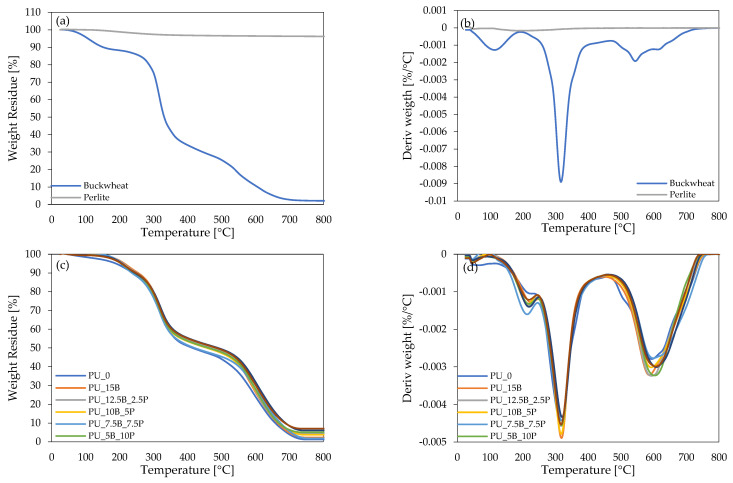
Thermogravimetric (TGA) and derivative thermogravimetry (DTG) results obtained for (**a**,**b**) buckwheat hulls and perlite fillers and (**c**,**d**) polyurethane composite foams.

The thermal stability of buckwheat and perlite modifiers and polyurethane composites was evaluated by thermogravimetric analysis (TGA) and thermogravimetric derivative (DTG). The results obtained during the experiment are presented in Figure 10a–d and Table 2.

Regarding the buckwheat hulls filler, three stages of thermal decomposition can be observed. This is most likely related to the high amounts of lignin (>30 wt.%), cellulose (>30 wt.%), and hemicellulose (>10 wt.%) in buckwheat husks [23]. The first stage occurred at approximately 100 °C and was most likely related to the evaporation absorbed by the filler moisture. The second stage of thermal decomposition, with a maximum rate of approximately 316 °C, was associated with the degradation of hemicellulose and cellulose. The final, third stage of the thermal degradation of buckwheat filler, with a maximum rate of approximately 544 °C, was related to the thermal decomposition of lignin [23,24]. In the case of the perlite modifier, it can be observed that perlite undergoes minimal temperature decomposition. Its weight loss at 800 °C is only approximately 3.8%. In the case of PU composites, their thermal stability is well illustrated by the data of individual mass losses and char residues at 600 °C, presented in Table 3. In the case of the introduction of both additives in the form of waste biomass and volcanic mineral, the thermal stability of the composites significantly improved compared to unmodified foam. The mass losses determined occurred at higher temperatures, which proves greater resistance to high temperatures after the introduction of the tested flame retardants. This is particularly visible in the case of determining the temperature at a 50% weight loss of composites, where, for example, for foams with a high content of perlite, an increase in stability by almost 20% was noted compared to the reference foam. In addition, all modified foams were characterized by a much higher char residue at 600 °C, which also proves the positive effect of flame retardants on thermal stability.

### 5.5. Thermal Conductivity

Because rigid PU foams are mainly used in the construction industry as insulating materials, one of the most important performance parameters of these materials is low thermal conductivity (λ). Thermal conductivity depends primarily on the content of closed cells, cell size, and apparent density [25,26]. The test results presented earlier show that the flame retardants used had a significant impact on the structure parameters of the obtained foams; therefore, their insulating properties after modification were also tested. Based on the results in Table 4, it can be observed that as a result of the introduction of modifiers, the heat transfer coefficient changed, which was a consequence of the change in the structure of the composites as a result of the introduction of flame retardants, which was manifested primarily by changes in the density and porosity of the foams. All modified foams were characterized by slightly higher thermal conductivity coefficients compared to the reference foam at each measurement temperature. This was, of course, due to the uneven structure, the presence of open pores, and the increasing share of the skeletal phase (ribs between the cells). The skeleton had a much higher thermal conductivity than the gaseous component; thus, the greater the proportion of the skeleton, the greater the thermal conductivity of the porous material. Similar observations were reported by Kurańska et al. [27], which showed that despite the high content of closed cells, the thermal conductivity coefficient for samples modified with basalt waste increased in the amount of 3–40% by weight. However, the value of thermal conductivity (up to 0.030 (W/(m·K)) was within the acceptable range from an application point of view [28]. An analogous dependence was also noted in the presented studies.

### 5.6. Burning Behavior

**Figure 11:**
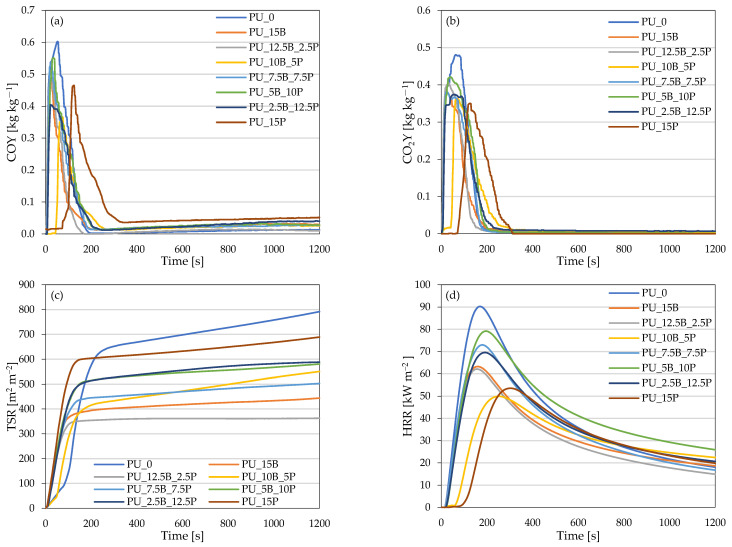
The results of (**a**) an average yield of CO, (**b**) an average yield of CO_2_, (**c**) total smoke release, and (**d**) peak of heat release rate.

The burning behavior of composite polyurethane foams was assessed using a cone calorimeter. The ignition time (IT), total heat release (THR), total smoke release (TSR), average yields of CO and CO_2_ (COY and CO_2_Y), and maximum average rate of heat emission (MAHRE) were assessed and are summarized in Table 5 and Figure 11a–d.

When compared with the reference foam PU_0, it can be observed that the addition of all modifiers influenced the burning behavior parameters. When it comes to the ignition time (IT), the addition of modifiers increased its value from 6 s for the unmodified foam to 8 s for PU_15B and PU_12.5B_2.5P, 9 s for PU_7.5B_7.5P, 10 s for PU_15P, 12 s for PU_5B_10P and PU_2.5B_12.5P, and 14 s for 10B_5P. This means that the addition of the fillers delayed the ignition of the composite. When analyzing the influence of the used fillers on total heat release (THR), a decrease in the values of this parameter was observed. A decrease in the values of THR parameter was observed from 27.2 MJ m^−2^ for the reference foam to even 20.9 MJ m^−2^ for PU_12.5B_2.5P, the lowest value. In the case of total smoke release (TSR) presented in Figure 11c, a decrease in the values of this indicator was also observed when compared with the unmodified foam PU_0. The values of the TSR parameter decreased from 792 m^2^ m^−2^ for the reference foam to 689, 588, 581, 550, 503, 444, and 363 m^2^ m^−2^ for PU_15P, PU_2.5B_12.5P, PU_5B_10P, PU_10B_5P, PU_7.5B_7.5P, PU_15B, and PU_12.5B_2.5P, respectively.

The addition of used modifiers also influenced the released gases. Based on the data shown in Table 5, it can be observed that when compared with the unmodified foam PU_0 (CO—1.031 kg kg^−1^; CO_2_—4.169 kg kg^−1^), the addition of all modifiers decreased the average yields of CO and CO_2_ of the composite foams. The PU_2.5B_12.5P foam was characterized by a higher average yield of CO (0.642 kg kg^−1^) than the other modified foams, while the other values oscillated between 0.220 and 0.350 kg kg^−1^. The average yield of CO_2_ also had lower values for modified polyurethane foams than for unmodified foams. The values of this parameter decreased from 4.169 kg kg^−1^ and ranged between 3.19 and 3.88 kg kg^−1^ for foams with added modifiers. The lowest values of the average yields of both CO and CO_2_ were observed in foam PU_12.5B_2.5P and they were 0.229 kg kg^−1^ (CO) and 3.198 kg kg^−1^ (CO_2_). The maximum average rate of heat emission is an important index when it comes to predicting large-scale fire development. The values of this parameter were also lower for modified foams when compared with the reference one. The value of MAHRE for PU_0 was 90.2 kW m^−2^, while the values of the foams with the addition of modifiers were in the range of 49.9 to 79.2 kW m^−2^. The lowest MAHRE value (49.9 kW m^−2^) was achieved by foam PU_10B_5P. Based on the above results, it can be observed that the incorporation of buckwheat/perlite modifiers improved the burning behavior of composite foams.

## 6. Conclusions

Rigid PU foams were modified with a flame-retardant system consisting of a component derived from waste biomass—buckwheat hulls and a volcanic mineral—perlite powder in a total amount of 15 parts by weight per 100 parts of polyol. The impact of flame retardants was examined on selected properties of PUR composites, such as structure, physical and mechanical properties (density, compressive strength, flexural strength, hardness), insulating properties (thermal conductivity), thermal properties (temperature of thermal decomposition stages), and flame-reducing properties (ignition time, total heat release, total smoke release, average CO and CO_2_ yield, maximum average heat release rate). Based on the presented test results, it can be concluded that the introduction of selected fillers into the rigid PU foam significantly affected the properties of the obtained composites. These additives caused a change in the morphology of the obtained foams—the structure became more heterogeneous with a greater number of open pores, which resulted in changes in density and hardness and a slight deterioration in compression or flexural strength. The foams obtained with the use of flame retardants were also characterized by reduced hydrophobicity, particularly those with a higher mass share of buckwheat hulls. Studies have also shown that changes in structure caused by the addition of an additional component increase thermal conductivity. Buckwheat hulls and perlite worked very well as flame retardants; it can be observed that the addition of buckwheat/perlite modifiers improved both the thermal stability and the burning behavior of composite foams.

## Figures and Tables

**Table 1:** The flame retardants added to the foam during synthesis in parts by weight per 100 parts of polyol.

Sample	Weight of Buckwheat Hulls	Weight of Perlite
PU_0	0	0
PU_15B	15	0
PU_12.5B_2.5P	12.5	2.5
PU_10B_5P	10	5
PU_7.5B_7.5P	7.5	7.5
PU_5B_10P	5	10
PU_2.5B_12.5P	2.5	12.5
PU_15P	0	15

**Table 2:** Effect of buckwheat hulls and perlite on the structural parameters of rigid PU foams.

Sample	Cell Size Distribution [µm]	Wall Thickness [µm]
PU_0	493–722	54 ± 5
PU_15B	421–795	53 ± 8
PU_12.5B_2.5P	564–911	56± 6
PU_10B_5P	471–856	57 ± 5
PU_7.5B_7.5P	448–826	60 ± 6
PU_5B_10P	391–707	62 ± 7
PU_2.5B_12.5P	343–1122	62 ± 6
PU_15P	382–1205	63 ± 7

**Table 3:** The results of the thermogravimetric analysis of rigid PU foams.

Sample	T_5_ [°C]	T_10_ [°C]	T_50_ [°C]	Char Residues (at 600 °C)
PU_0	189	238	414	23.98
PU_15B	213	254	474	26.33
PU_12.5B_2.5P	212	251	470	27.05
PU_10B_5P	205	246	456	27.41
PU_7.5B_7.5P	204	245	440	28.16
PU_5B_10P	205	244	462	27.97
PU_2.5B_12.5P	208	245	489	31.75
PU_15P	205	248	484	30.17

**Table 4:** Thermal conductivity coefficients at temperatures 10, 20, and 40 °C.

Sample		λ	
		[W/m·K]	
	10 °C	20 °C	40 °C
PU_0	0.02481	0.02610	0.02908
PU_15B	0.03011	0.03160	0.03462
PU_12.5B_2.5P	0.03059	0.03199	0.03510
PU_10B_5P	0.02928	0.03067	0.03361
PU_7.5B_7.5P	0.02761	0.02893	0.03157
PU_5B_10P	0.02775	0.02854	0.03189
PU_2.5B_12.5P	0.02713	0.02846	0.03144
PU_15P	0.02887	0.03059	0.03361

**Table 5:** Burning behavior parameters of composite polyurethane foams.

Sample	IT [s]	THR [MJ m^−2^]	TSR [m^2^ m^−2^]	COY [kg kg^−1^]	CO_2_Y [kg kg^−1^]	MARHE [kW m^−2^]
PU_0	6	27.2	792	1.031	4.169	90.2
PU_15B	8	22.4	444	0.347	3.696	63.2
PU_12.5B_2.5P	8	20.9	363	0.229	3.198	62.1
PU_10B_5P	14	24.5	550	0.250	3.677	49.9
PU_7.5B_7.5P	9	24.4	503	0.338	3.559	73.0
PU_5B_10P	12	25.6	581	0.334	3.874	79.2
PU_2.5B_12.5P	12	24.8	588	0.642	3.303	69.5
PU_15P	10	23.8	689	0.3395	3.600	53.6

## Data Availability

Not applicable.
